# The Therapeutic Community: A Unique Social Psychological Approach to the Treatment of Addictions and Related Disorders

**DOI:** 10.3389/fpsyt.2020.00786

**Published:** 2020-08-06

**Authors:** George De Leon, Human F. Unterrainer

**Affiliations:** ^1^ Department of Psychiatry, NYU School of Medicine, New York City, NY, United States; ^2^ Center for Integrative Addiction Research (CIAR), Grüner Kreis Society, Vienna, Austria; ^3^ University Clinic for Psychiatry and Psychotherapeutic Medicine, Medical University Graz, Graz, Austria; ^4^ Institute for Religious Studies, University of Vienna, Vienna, Austria

**Keywords:** community as method, overview, group therapy, substance use disorder, therapeutic community

## Abstract

The evolution of the contemporary Therapeutic Community (TC) for addictions over the past 50 years may be characterized as a movement from the marginal to the mainstream of substance abuse treatment and human services. TCs currently serve a wide array of clients and their diverse problems; through advances in research in treatment outcomes, the composition of staff has been reshaped, the duration of residential treatment has been reduced, the treatment goals have been reset and, to a considerable extent, the approach of therapy itself has been modified. An overview of the TC as a distinct social-psychological method for treating addiction and related disorders is provided by this paper. Included in this is a focus on the multifaceted psychological wounds that consistently show a strong association with addiction and thereby require initiating a recovery process characterized by life-style and identity changes.

## Introduction

We intend here to give a brief overview of the development of the therapeutic communities (TCs) for the treatment of addictions. After a brief historical introduction (pt 1), the core dimensions of the TC treatment approach will be introduced and the characteristic peculiarities will be discussed (pt 2). Based on this, we will describe the “community as method” approach in more detail and show different approaches to empirically depict the change process that the patients go through during their stay in the TC (pts 3–5). In line with this, the state of research regarding the possible change processes taking place in the TC will be summarized (pt 6) and possible other forms of application of TCs for special populations and settings will be discussed (pt 7). Lastly, we conclude by summarizing the TC key elements, also in comparison to other therapeutic approaches (pt 8).

## The Evolution of the TC

According to DeLeon (1) “the idea of therapeutic community recurs throughout history, implemented in different incarnations. Communities that teach, heal, and support, appear in religious sects and utopian communes, as well as in spiritual, temperance, and mental health reform movements.” (p.11). In correspondence to this, indirect influences on TC concepts, beliefs, and practices can be found in religion, philosophy, psychiatry, and the social and behavioral sciences. Thereby, early prototypes of communal healing and support can be traced back to classical antiquity. Remarkably, there are two elements in ancient medical texts that can also be applied to modern TCs for addictions: 1) the mental illness (or the disease of the soul) manifests itself as a disease of the whole person and is characterized in particular by problems with self-control on the behavioral and emotional level and 2) the healing of the disease (or the soul) happens through the involvement of a community or group. Then, as now, violations of the rules of the community were sanctioned or had negative consequences for the individual. In this sense, the group also determines the type and extent of the sanctions, which in the case of serious violations, especially against the integrity of the group, can also mean expulsion from the community (2).

Although the TC for addictions has been influenced by numerous sources, both current and historical teachings can be found herein, the actual term “therapeutic community” can be considered modern. This was first used to describe psychiatric TCs in Great Britain during the 1940s (3). However, it is unclear, how these first TCs (for general psychiatric patients) have influenced the development of the TCs focusing on addictions, which began in the United States (4). In North America, Charles Dederich, as a former alcoholic himself and member of “Alcoholics Anonymous”, founded one of the first self-help groups for opiate addictions in 1958 named “Synanon”. Primarily, he was inspired by the works of the writer and philosopher R. W. Emerson and a religious organization called “The Oxford Group”, which saw itself as a moral antipode to international armament. This group was also influenced by “Alcoholics Anonymous” and their 12-step method of treating alcohol addiction. So-called Synanon houses and Synanon villages developed, in which former addicts renounced their old way of life, concentrating instead on the present moment and communal work, which was based on values such as truth and sincerity (5).

In Europe the first TCs shaped by American models were founded in the mid-1960s. A self-help group called “Release” was setup in England in 1967. As a result of the success of “Release” TCs were independently developed in several countries across Europe from the 1960s and 1970s (5). As illustrated, for example, by Cortini, Clerici, and Carrà (6), today we can certainly speak of a unique evolutionary strand of the TC movement in Europe. Furthermore, the authors rightly argued that a comparison of both the European and the American treatment routes could contribute to a more differentiated discussion of the TC treatment concepts in general as well as guide their further development. Although different variations of TCs have developed in the United States as well as in Europe independently from each other, they still share some key core elements which will be further characterized now.

## The TC Perspective

Comprehensive accounts of the TC theory, model, and method are contained in De Leon (1, 7). The TC theory, “community as method” shapes its program model and its unique approach. The paradigm is comprised of four interconnected views of substance use disorder and how the individual, process of recovery, and living healthy are defined.

### View of the Disorder

The abuse of drugs is considered a comprehensive disorder affecting the whole person and many, if not all, parts of functioning. It is evident that those suffering from drug abuse have problems not only with cognition and behavior, but also mood disturbances (8). The substance abusing individual’s thoughts may be classed as unrealistic or even disorganized, their values are mixed up, antisocial or even nonexistent (9, 10). All too often they suffer from deficits in comprehension, writing, reading, and so-called “marketable skills” (11). Spiritual struggles, or even moral problems, are consistently apparent whether expressed in psychological or existential terms (12). Thus, it can be argued that the problem is with the individual and not the substance abused; in other words, addiction can be seen as a symptom rather than the essence of their disorder (13). This perspective may also be one of the main characteristics of the TC and one of the major differences between the TC model and standard psychiatric inpatient treatment, which is much more based on symptom-oriented diagnostic systems such as the International Classification of Diseases in 11^th^ revision [ICD 11; (14)] or the Diagnostic and Statistical Manual of Psychiatric Disorders Version 5 [DSM 5; (15)].

Accordingly, in terms of an attachment based therapeutic approach, it can be said that the TC tries to break the bond to a substance and instead direct the patient toward forming a bond to the community. Thereby, the TC can serve as an attachment figure, acting as a safe haven in which one can enter, but also as a secure base from which one can start again into a new (drug-free) life (16). While clinical evidence suggests the important role of the community for the functioning of affect regulation (17), some additional support comes from a neuro-evolutionary perspective e.g., through the “social baseline” model, which proposes “that social species are hard-wired to assume relatively close proximity to conspecifics, because they have adopted social proximity and interaction as a strategy for reducing energy expenditure relative to energy consumption” [(18), p. 19; see also (19), for a more general discussion].

### View of the Person

In TCs, rather than classifying individuals according to their patterns of drug abuse, they are instead delineated along degrees of “psychological dysfunction” and “social deficits”. Additionally, many residents in TCs have manifested vocational and educational problems; society’s mores are either ignored or totally avoided. These residents are often from a socially depressed sector. A better term for their TC experience is “habilitation”, the development of a social, productive, and “conventional” lifestyle for the first time. However, among residents from more advantaged backgrounds, the term “rehabilitation” is judged more appropriate since it emphasizes a return to a rejected lifestyle previously lived and known. Despite apparent differences in social background, psychological problems, or drug preferences most individuals admitted to TCs share profound clinical characteristics that center around antisocial dimensions or immaturity (**Table 1**). Whether they precede or follow serious involvement with drugs, these characteristics are commonly observed to correlate with substance dependency. Crucially, in TCs, a change for the better in these characteristics is thought to be essential for long-term recovery (1).

**Table 1:** Typical behavioral, cognitive, and emotional characteristics of substance abusers in therapeutic communities.

Low tolerance for all forms of discomfort and delay of gratification
Problems with authority
Inability to manage feelings (particularly hostility, guilt, and anxiety)
Poor impulse control (particularly sexual or aggressive impulses)
Poor judgment and reality testing concerning consequences of actions
Unrealistic self-appraisal regarding discrepancies between personal resources and aspirations
Prominence of lying, manipulation, and deception as coping behaviors
Personal and social irresponsibility (e.g. inconsistency or failures in meeting obligations)
Marked deficits in learning and in marketable and communication skills

From the TC perspective, for recovery to occur a change in lifestyle, in addition to social and personal identity, is considered vital. Thus, the main psychological goal of treatment is an attempt to change negative patterns of thinking, behavior, and feeling that predisposes an individual to drug use; meanwhile the main social goal is to develop skills, attitudes, and instill values necessary for a responsible, drug-free lifestyle. Stable recovery, however, is dependent on a successful integration of these psychological and social goals. Without insight behavioral change is unstable; however, without lived experience mere insight is insufficient. Several key assumptions underlie the recovery process in the TC (1).

#### Motivation

Recovery depends on pressures, both positive and negative, to change. For example, certain people might seek help due to stressful external pressures; others may be moved by more intrinsic factors. For everyone, however, sticking to a treatment program requires a continual internal motivation to change. Thus, some elements of the treatment approach are designed to either sustain motivation or enable early detection of signals that the subject might terminate treatment prematurely (1).

#### Self-Help and Mutual Self-Help

In practical terms treatment is not provided per se; rather, it is provided to all individuals in the TC through the daily regimen of groups, seminars, work, recreation and meetings, and its staff and peers. The efficacy of these elements, however, depends on the individual: they must engage fully in the treatment regimen for best outcomes. In self-help recovery the individual must make the main contribution to his/her change process. By contrast, in mutual self-help the primary messages of personal growth, “right living”, and recovery are mediated by peers through discourse and sharing experiences in groups, providing examples as role models, and acting as encouraging, supportive friends in daily interactions (1).

#### Social Learning

Lifestyle changes occur in a social context. Negative behavioral attitudes, patterns, and roles, in general, are not acquired in isolation, nor can they be ameliorated in isolation. Thus, this presupposition is the basis for the view that a peer community can facilitate recovery. Social responsibility as a role is learned by acting the role within a community of one’s peers (1).

## View of Right Living

TCs adhere to certain values, precepts, and a social perspective that guides and reinforces recovery. For instance, there exist community sanctions that address antisocial attitudes and behavior: emphasis is also placed on changing the negative values of irresponsible or exploitative sexual conduct, in jails, negative peers or “the streets”. Positive values, by contrast, are given a positive emphasis as being essential to both social learning and personal growth. These values include such concepts as truth and honesty (both in word and deed), a strong work ethic, a feeling of responsibility for others (e.g. being one’s brother’s or sister’s keeper), a sense of achievement and that all rewards have been earned, self-reliance, personal accountability, community involvement, and social manners. The values of “right living” are reinforced constantly in various informal and formal ways (e.g. signs, seminars, in groups, and community meetings) (1).

In order to counter the concerns of critics, attempts have been made to date to scientifically prove the effectiveness of the treatment concept (20). However, the therapeutic concept of the TC is being questioned due to the lack of randomized clinical studies with regard to the therapeutic success. Despite these criticisms, “community as method” can still be seen as the top principle of the TC, both in terms of the treatment and the research into change processes in the TC. This method will now be explained in more detail (21).

## TC Approach: Community as Method

The approach of TC can be summarized by the phrase “community as method”. The definition of community as method offered by theoretical writings is as follows: The purposive use of the community to teach individuals to use the community to change themselves. Thus, the fundamental assumption that underlies the concept of community as method is: individuals obtain maximum educational and therapeutic impact when they engage in, and learn to use, all of the diverse elements of the community as tools for self-change. Therefore, “community as a method” means that the community is both context and mediator for individual change and social learning. Its membership establishes expectations or standards of participation in the community. It assesses how individuals are meeting these expectations and respond to them with strategies that promote continued participation (1).

## Community, the Individual, and the Process of Change

Everyone uses the expectations and context of their community to change and learn. Living up to the expectations of their community requires that an individual continually change their behaviors, attitudes, and emotional management. Conversely, avoidance of, or difficulties in living up to community expectations can also result in an individual’s growth through continual self-examination, re-motivation to engage in trial and error learning, and re-committing to the process of change. Thus, the drive to cohere to what the community expects for participation compels residents to pursue personal goals of psychological growth and socialization. The whole process can be summed up in the phrase: if you participate, then you will change (1).

## TC Research: Direct Evidence for Social and Psychological Changes

A considerable scientific knowledge base has been developed over the past four decades, with the addition of follow-up studies on thousands of individuals treated in TCs. The most extensive body of research bearing on the efficacy of TC programs involving addiction has been collected from numerous field outcome studies. All of these studies utilized similar longitudinal designs that followed admissions to TCs during treatment and 1–5 years (and in one study up to 12 years) after leaving the index treatment. These studies consistently show that TC admissions have poor profiles with regard to severity of substance use, psychological symptoms and social deviance. The striking replicability across studies has left little doubt as to the reliability of the overarching conclusion: There is a consistent correlation between treatment retention in TCs and positive post-treatment outcomes. This conclusion is additionally supported in the smaller number of controlled and comparative studies involving TC programs [for enhanced reviews of the TC outcome literature in North America, see (21); and internationally, see (20)].

## Indirect Evidence Based Social Psychological Principles and Practices Are Embedded Within Community as Method

The TC for addictions emerged practically, outside both mainstream mental health and social science. Nevertheless, a unique theoretical social learning approach has evolved, captured in the phrase “community as method”. The latter, however, contains elements and practices that are familiar and supported by abundant social–psychological and behavioral research outside of TCs (**Table 2**). Similarly, behavioral training and social learning principles are obvious, e.g. vicarious learning, the training, and acquisition of social roles and social reinforcement. As discussed elsewhere, these principles are naturally mediated by the context of community living (1).

**Table 2:** Indirect evidence: examples of TC program and practice elements that are evidence-based in the behavioral and social-psychological research literature.

*Peer Tutoring*
TC: Mutual self-help grounded in peers as role models and mentors.
*Therapeutic Alliance*
TC: Affiliation and participation in the program depends upon the relationship between the individual and the community
*Motivational Enhancement*
TC: Group process focuses individuals on problem identification and encourage desire to change.
*Behavior Modification*
TC: System of verbal correctives and affirmations as well as social sanctions and privileges for facilitating behavioral change.
*Goal Attainment*
The program plan focuses on incremental learning, defined by specific stage and phase outcomes leading to program completion.

TC, therapeutic communities.

Overall, the weight of the direct evidence from all sources (e.g., multiple sources of outcome research in North America which includes single program controlled studies, cost–benefit studies, meta-analytic statistical surveys, and multi-program field effectiveness studies) supports the conclusion that the TC is both a cost-effective and therapeutically effective treatment for certain substance abuser subgroups, particularly those with severe drug use, social and psychological problems. This conclusion is supported by considerable indirect evidence from social psychological principles and practices that are inherent within community as method. Other strategies that are informed by evidence can be incorporated to enhance rather than substitute for community as method, the primary approach (1).

TC model was developed further or adapted to different circumstances or patient groups. These changes will be briefly explained below.

## TC Applications to Special Populations and Settings

The traditional TC model described herein is in actuality the prototype of a variety of TC oriented programs. Today TC modality largely consists of a wide range of programs that serve a variety of patients who use diverse drugs and who, in addition to their chemical abuse, present with complex psychological and social problems. Clinical requirements as well as client differences, in addition to the reality of funding, have encouraged the development of modified residential TC programs that offer shorter planned durations of stay (3, 6, and 12 months) as well as TC-oriented outpatient ambulatory models and day treatments. Correctional facilities, medical and mental hospitals, and community residences and shelters, having become overwhelmed with alcohol and drug abuse problems, have implemented TC programs within these settings (11).

Most community-based traditional TCs have either incorporated new interventions or expanded their social services to address the diverse needs of their members. These changes and additions include specific primary healthcare geared toward individuals with AIDS or who are HIV-positive, family services, relapse prevention training, aftercare services specifically for special populations such as substance-abusing inmates leaving prison treatment, mental health services, components of 12-step groups, and other evidence-based practices (e.g., cognitive-behavioral therapy, motivational interviewing). These modifications and additions enhance, but are not intended as a substitute for, the fundamental TC approach: Community as method. Research literature documents the effectiveness and cost-effectiveness of modified TCs for special populations such as homeless and mentally ill chemical abusers, those in criminal justice settings and adolescents (1, 22–26).

## Concluding Remarks

The fundamental, primary foundation for the TC program model, its distinctive methodology, community as method, and its longer than usual treatment duration is the recovery perspective. Fundamentally, multi-dimensional (“whole person”) change necessarily requires a multi-interventionist approach that is sustained for a sufficient amount of time (1).

The TC for addictions is arguably one of the first formal treatment paradigms that is overtly recovery oriented. Although Alcoholics Anonymous and similar programs, focused on an approach of mutual self-help, facilitate recovery these programs differ from TC by representing their service as support rather than treatment. Meanwhile, pharmacological treatment paths, such as methadone, have as their putative treatment objective the outright elimination or, at the very least, reduction of the abuse of opiates. Empirically based approaches to behavior, such as motivational enhancement (MET), cognitive behavioral therapy (CBT), and contingency contracting, focus upon reducing the abuse of the targeted drug. From the TC’s perspective, however, the main goal of treatment is “recovery” which is broadly defined as identity and lifestyle changes. These changes involve abstaining from the illicit use of narcotics (and other drugs), the total elimination of social deviance and the development of positive social values and appropriate behavior (1). Thus, the mission, and that which distinguishes TC from other treatment paths, is promoting recovery and encouraging living right.

## Author Contributions

GDL wrote the draft of the manuscript. HU read the manuscript and made some critical comments. All authors contributed to the article and approved the submitted version.

## Conflict of Interest

The authors declare that the research was conducted in the absence of any commercial or financial relationships that could be construed as a potential conflict of interest.
